# The high mobility group protein HMO1 functions as a linker histone in yeast

**DOI:** 10.1186/s13072-016-0062-8

**Published:** 2016-03-30

**Authors:** Arvind Panday, Anne Grove

**Affiliations:** Department of Biological Sciences, Louisiana State University, Baton Rouge, LA 70803 USA

**Keywords:** Chromatin, High mobility group protein, Histone H1, Chromatin immunoprecipitation, Double-strand break repair, Hho1p

## Abstract

**Background:**

Eukaryotic chromatin consists of nucleosome core particles connected by linker DNA of variable length. Histone H1 associates with the linker DNA to stabilize the higher-order chromatin structure and to modulate the ability of regulatory factors to access their nucleosomal targets. In *Saccharomyces cerevisiae*, the protein with greatest sequence similarity to H1 is Hho1p. However, during vegetative growth, *hho1∆* cells do not show any discernible cell growth defects or the changes in bulk chromatin structure that are characteristic of chromatin from multicellular eukaryotes in which H1 is depleted. In contrast, the yeast high mobility group (HMGB) protein HMO1 has been reported to compact chromatin, as evidenced by increased nuclease sensitivity in *hmo1∆* cells. HMO1 has an unusual domain architecture compared to vertebrate HMGB proteins in that the HMG domains are followed by a lysine-rich extension instead of an acidic domain. We address here the hypothesis that HMO1 serves the role of H1 in terms of chromatin compaction and that this function requires the lysine-rich extension.

**Results:**

We show here that HMO1 fulfills this function of a linker histone. For histone H1, chromatin compaction requires its basic C-terminal domain, and we find that the same pertains to HMO1, as deletion of its C-terminal lysine-rich extension renders chromatin nuclease sensitive. On rDNA, deletion of both HMO1 and Hho1p is required for significantly increased nuclease sensitivity. Expression of human histone H1 completely reverses the nuclease sensitivity characteristic of chromatin isolated from *hmo1∆* cells. While chromatin remodeling events associated with repair of DNA double-strand breaks occur faster in the more dynamic chromatin environment created by the *hmo1* deletion, expression of human histone H1 results in chromatin remodeling and double-strand break repair similar to that observed in wild-type cells.

**Conclusion:**

Our data suggest that *S. cerevisiae* HMO1 protects linker DNA from nuclease digestion, a property also characteristic of mammalian linker histone H1. Notably, association with HMO1 creates a less dynamic chromatin environment that depends on its lysine-rich domain. That HMO1 has linker histone function has implications for investigations of chromatin structure and function as well as for evolution of proteins with roles in chromatin compaction.

## Background

Genomic DNA is packaged into nucleosomes by association with core histones, which are among the most highly evolutionarily conserved proteins. The linker DNA that separates these nucleosome core particles may associate with histone H1, much more heterogeneous proteins that condense the polynucleosome fiber [1, 2]. H1 proteins typically contain a short N-terminus, a central globular domain, and a basic C-terminal domain, and mammalian cells encode multiple somatic isoforms. H1 binds the DNA that enters and exits the nucleosome and bends it as a first step toward formation of a compact structure. This binding is mediated by the globular domain, however, the chromatin compaction function of H1 requires its basic C-terminal extension, which organizes the linker DNA; the C-terminal domain operates as an intrinsically disordered protein with folding coupled to DNA binding and with important roles in establishing residence time on the nucleosome [3–8]. Interaction of H1 with linker DNA manifests as an increased resistance to digestion by micrococcal nuclease (MNase) [9].

The extensive compaction imposed by nucleosomes and linker histones is generally a barrier to events such as DNA repair and gene transcription, and covalent modification of histones as well as nucleosome remodeling operates together to facilitate access to required DNA-dependent machineries. For example, early events following induction of DNA double-strand breaks include phosphorylation of histone H2A (H2AX in mammalian cells), which is associated with recruitment of proteins to repair foci [10]. More recently, ubiquitylation of human H1 was also implicated in recruitment of repair factors [11]. Consistent with a function in compacting chromatin, binding of histone H1 has generally been associated with repression of transcription and DNA repair [12, 13].

Acting in opposition to H1, mammalian high mobility group (HMGB1) proteins contain two HMG domains (box A and box B) followed by an acidic C-terminal extension. With binding sites for H1 and HMGB1 partially overlapping, likely resulting in mutually exclusive interactions with the DNA entry/exit points on the nucleosome, HMGB1 proteins have been shown to induce a less stable chromatin structure [14–17]. HMGB proteins are ~ 10 times less abundant than H1, more mobile, and bind with lower affinity. Like H1, HMGB1 bends DNA, but the acidic C-terminus lowers DNA binding affinity for linear DNA and confers preferred binding to pre-bent or distorted DNA [18, 19]. The C-terminal extension has also been reported to interact directly with the N-terminal tail of histone H3 [20]. Exchange of H1 for HMGB1 and vice versa is likely facilitated by the fast on/off rates characteristic of both proteins [14].

Yeast was long thought to lack histone H1 until sequencing identified Hho1p as having the greatest sequence similarity to H1 [21]. Hho1p has a different modular organization, with the H1-like globular domain followed by a short basic linker and a second globular domain. Moreover, during vegetative growth the absence of Hho1p does not result in any apparent phenotype or notable change in bulk chromatin structure, as evidenced by changes in MNase sensitivity [21, 22]. Evidence is also accumulating that Hho1p has little overall effect on transcription, as inactivation of *hho1* only results in differential expression of <1 % of genes [23–25]. Roles of Hho1p in transcription may instead be due to more subtle functions, such as a contribution to silencing and barrier element activity [26, 27]. Hho1p has also been reported to inhibit repair of DNA double-strand breaks by homologous recombination (but not non-homologous end-joining [28]); its roles in homologous recombination have been associated with yeast aging, a phenotype that may be linked to its contribution to formation of chromatin loops [29].

Yeast contains several HMGB proteins of which the single-HMG-domain proteins Nhp6A/B have been associated with changes in gene activity and chromatin structure, but no changes in bulk chromatin structure were seen in *nhp6A/B* mutant strains as measured by sensitivity to MNase digestion [30]. By contrast, deletion of HMO1 was reported to render chromatin hypersensitive to nuclease [31]. Genome-wide association of HMO1 with chromatin is variable; HMO1 is highly enriched at sites such as rDNA and genes encoding ribosomal proteins [32, 33], with lower occupancy at other sites. However, nearly 1000 genes were reported to have HMO1 occupancy at least twofold above background, consistent with the ability to detect changes in MNase sensitivity when examining bulk chromatin [32]. Consistent with its abundance at rDNA, HMO1 has been implicated in rDNA transcription and rRNA processing [34, 35]. Specialized functions in coordinating expression of rDNA and genes encoding ribosomal proteins in response to signaling by target of rapamycin (TOR) kinase have also been well established [36–38].

HMO1 has two globular box A and box B domains, of which only box B is a consensus HMG domain, followed by a C-terminal lysine-rich domain. The presence of a lysine-rich extension in HMO1 is unusual for HMGB proteins and likely to result in properties distinct from those characteristic of vertebrate HMGB1 proteins. In vitro, both box A and box B contribute to DNA binding [39], whereas the C-terminal domain is required for DNA compaction and in-phase DNA bending as well as for optimizing nuclear import [40–43]. We show here that HMO1 functions as a linker histone as evidenced by the observation that the more dynamic chromatin structure created by *hmo1* deletion is reversed by expression of human H1.

## Results and discussion

### The C-terminal domain of HMO1 is required for chromatin compaction

**Fig. 1:**
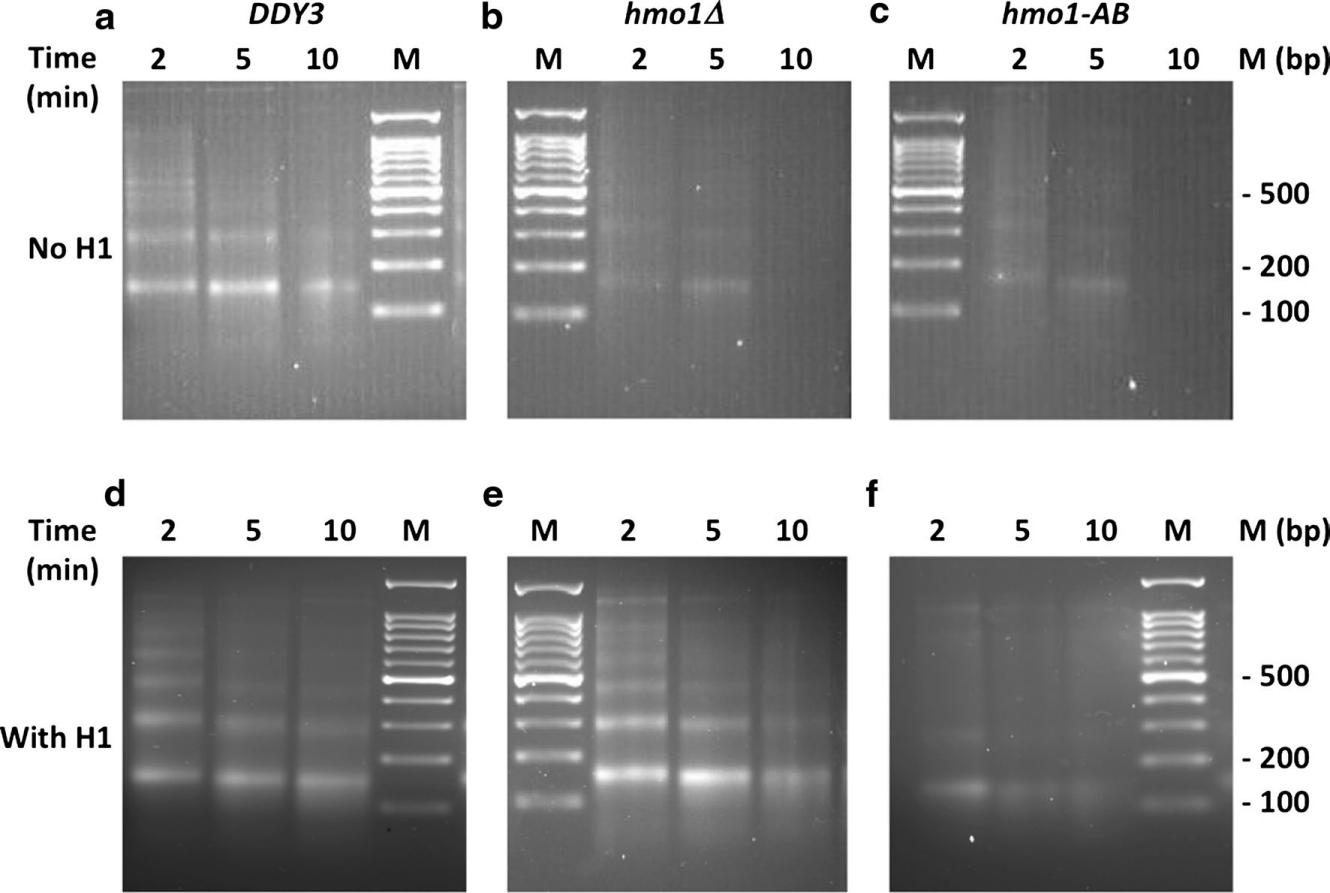
Resistance of chromatin to nuclease digestion requires linker histone H1 or HMO1 containing its lysine-rich extension. **a**–**c** MNase digestion of chromatin isolated from wild-type cells (*DDY3*), *hmo1∆*, and *hmo1*-*AB*, respectively. **d**–**f** MNase digestion of chromatin isolated from wild-type, *hmo1∆*, and *hmo1*-*AB* cells expressing human linker histone H1.2 under control of a strong, constitutive promoter. Nuclei were digested with 0.25 U/µl MNase for the time indicated. Nucleosomal DNA was purified and resolved by agarose gel electrophoresis and stained with ethidium bromide

To address whether the C-terminal domain of HMO1 participates in chromatin compaction in vivo during vegetative growth, as reflected in protection of linker DNA from nuclease digestion, we performed micrococcal nuclease (MNase) digestion of chromatin isolated from wild-type cells, *hmo1∆*, and *hmo1*-*AB* that expresses HMO1 truncated for its C-terminal tail [44]. MNase creates double-stranded cuts between nucleosomes, eventually resulting in predominantly DNA corresponding to the length of a mononucleosome (~146 bp). With time of incubation with MNase, chromatin from wild-type cells was depleted of larger DNA fragments while DNA corresponding to mono-nucleosomes accumulated (Fig. 1a). As expected, chromatin from *hmo1∆* cells was much more sensitive to digestion, and no DNA remained after 10-min incubation (Fig. 1b). Notably, chromatin from *hmo1*-*AB* cells was as hypersensitive to nuclease as *hmo1∆* cells (Fig. 1c), indicating that the ability to protect linker DNA requires the C-terminal domain of HMO1.

**Fig. 2:**
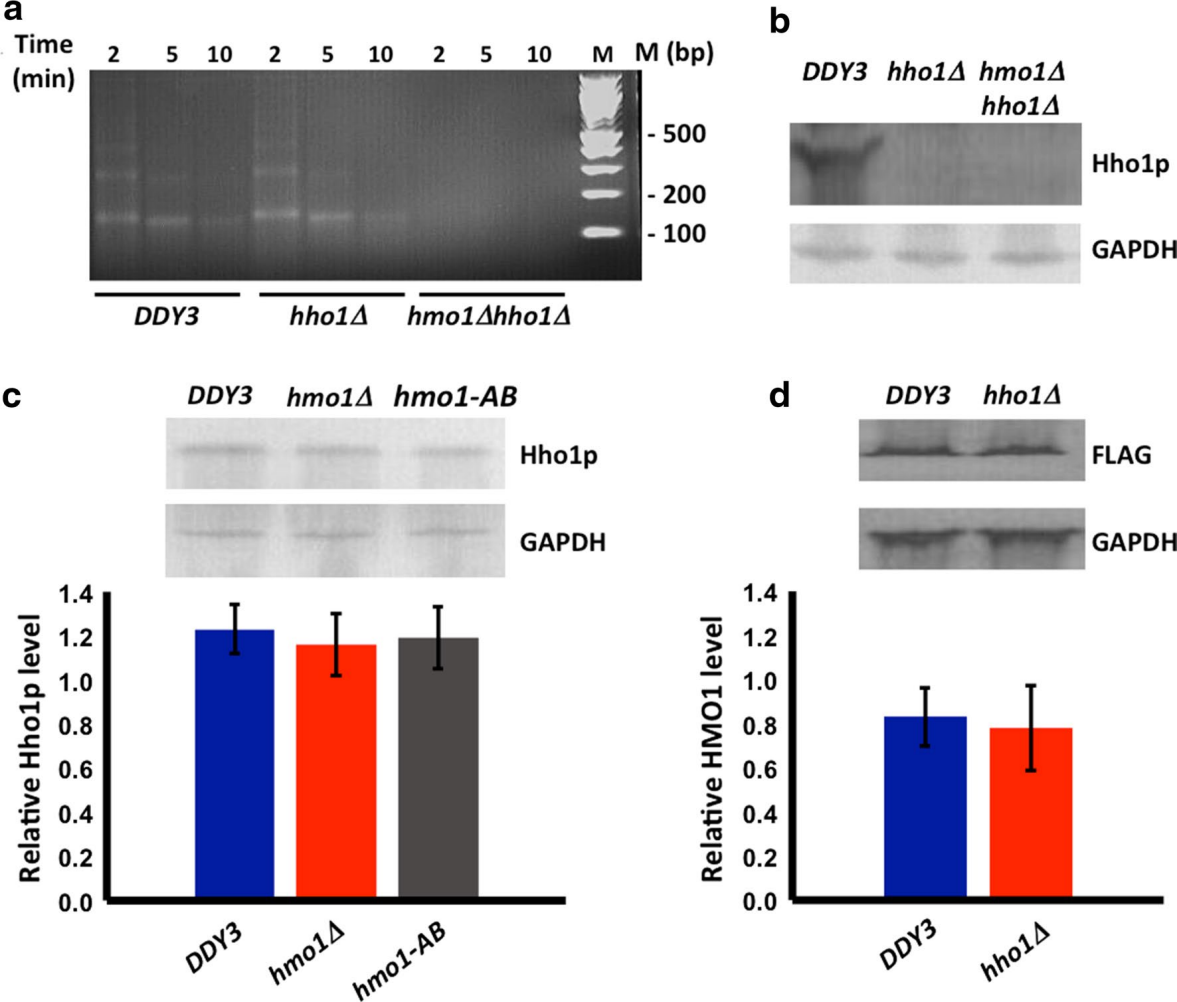
Effect of Hho1p on resistance of chromatin to nuclease digestion and on cellular content of HMO1. **a** MNase digestion of chromatin isolated from *DDY3*, *hho1∆*, and *hmo1∆ hho1∆*, respectively. Nuclei were digested with 0.25 U/µl MNase for the time indicated. Nucleosomal DNA was purified and resolved by agarose gel electrophoresis and stained with ethidium bromide. **b** Western blot of lysates from *DDY3*, *hho1∆*, and *hmo1∆ hho1∆* using antibody to Hho1p or GAPDH. GAPDH migrates with a Mw ~36 kDa, while Hho1p migrates with a Mw ~28 kDa. **c** Western blot of lysates from *DDY3*, *hmo1∆*, and *hmo1*-*ΑΒ* using antibody to Hho1p or GAPDH. Densitometric analysis of three separate blots from three independent experiments shown below. Relative level = Hho1p/GAPDH. **d** Western blot of lysates from *DDY3* and *hho1∆* using antibody to FLAG-tagged HMO1 or GAPDH. HMO1-FLAG migrates with a Mw ~35 kDa. Densitometric analysis of three separate blots from three independent experiments shown below. Relative level = FLAG/GAPDH. *Error bars* represent standard deviation

Vegetatively growing *hho1∆* cells were previously reported not to exhibit enhanced MNase sensitivity [21, 22]. To verify this phenotype under our experimental conditions and in the *DDY3* genetic background, we created an *hho1∆* strain as well as a strain in which both *hmo1* and *hho1* were inactivated and verified the absence of Hho1p by Western blot (Fig. 2b). As shown in Fig. 2a, inactivation of *hho1* does not result in altered sensitivity to MNase, as expected. However, DNA from cells in which both genes encoding HMO1 and Hho1p are inactivated were more sensitive to MNase digestion compared to *hmo1∆* cells. To address whether cellular levels of Hho1p and HMO1 change on inactivation of genes encoding the other protein, we performed Western blot. As shown in Fig. 2d, cellular content of HMO1-Flag is unaltered in the *hho1∆* strain; this is consistent with genome-wide analysis of gene expression in an *hho1∆* strain, in which *hmo1* was not differentially expressed [23], and it suggests that the unaltered MNase sensitivity of *hho1∆* cells is not due to compensatory *hmo1* expression. Conversely, cellular content of Hho1p is not affected on inactivation of *hmo1* or in cells expressing HMO1-AB (Fig. 2c).

**Fig. 3:**
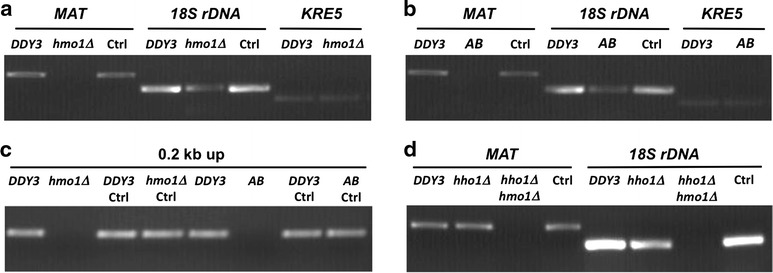
Resistance of chromatin to MNase digestion monitored at specific loci. **a**, **b** Amplification of DNA representing *MAT*, 18S rDNA, and *KRE5* after MNase digestion of chromatin isolated from wild-type cells (*DDY3*) and *hmo1∆* (**a**) or *hmo1*-*AB* (**b**). **c** Amplification of DNA using primers annealing 0.2 kb upstream of the HO cleavage site within the *MAT* locus from *DDY3*, *hmo1∆*, and *hmo1*-*AB*. **d** Amplification of DNA representing *MAT* and 18S rDNA from chromatin isolated from *DDY3*, *hho1∆*, and *hmo1∆hho1∆*. In all panels, *Ctrl* denotes chromatin from the identified strain not incubated with MNase. Data are representative of three repeats

Since association of HMO1 with the yeast genome is variable, with particular enrichment at sites such as rDNA and low or undetectable levels at other loci, we also probed specific DNA sites after MNase digestion using PCR. As shown in Fig. 3a, DNA at *MAT* and 18S rDNA (both loci at which HMO1 was amply detected [32]) was amplified as efficiently from DNA from wild-type *DDY3* cells exposed to MNase for 5 min as cells not incubated with MNase (Ctrl). By contrast, *MAT* DNA cannot be amplified from *hmo1∆* cells, whereas amplification of DNA representing 18S rDNA was less efficient in *hmo1∆*. At *KRE5*, where HMO1 was not abundant, equivalent amplification was observed in *DDY3* and *hmo1∆* cells. Cells expressing HMO1-AB deleted for the C-terminal tail featured the same pattern of DNA amplification as *hmo1∆*, validating the interpretation that the C-terminal extension is required for the observed resistance to MNase digestion (Fig. 3b). The inability to amplify DNA at the *MAT* locus after MNase digestion of DNA from *hmo1∆* or *hmo1*-*AB* was verified using primers that anneal 0.2 kb upstream of the cleavage site for the HO endonuclease within the *MAT* locus (Fig. 3c).

Association of Hho1p with genomic DNA has also been reported to be variable, with enrichment at rDNA [24]. Inactivation of *hho1* did not affect amplification of DNA from the *MAT* locus after MNase digestion, whereas no DNA was amplified using DNA from the *hmo1∆hho1∆* strain (Fig. 3d). Amplification of DNA from the *KRE5* locus after MNase digestion was equivalent for *DDY3*, *hho1∆* and *hmo1∆hho1∆* (data not shown). By contrast, MNase digestion of DNA from the *hho1∆* strain resulted in modestly reduced amplification of 18S rDNA, whereas MNase digestion of DNA from the *hmo1∆hho1∆* strain resulted in a failure to amplify 18S rDNA (Fig. 3d). The implication of this observation is that both HMO1 and Hho1p contribute to protection of this locus, a conclusion that is consistent with previous reports that both proteins associate with rDNA [24, 33, 35, 45, 46].

**Fig. 4:**
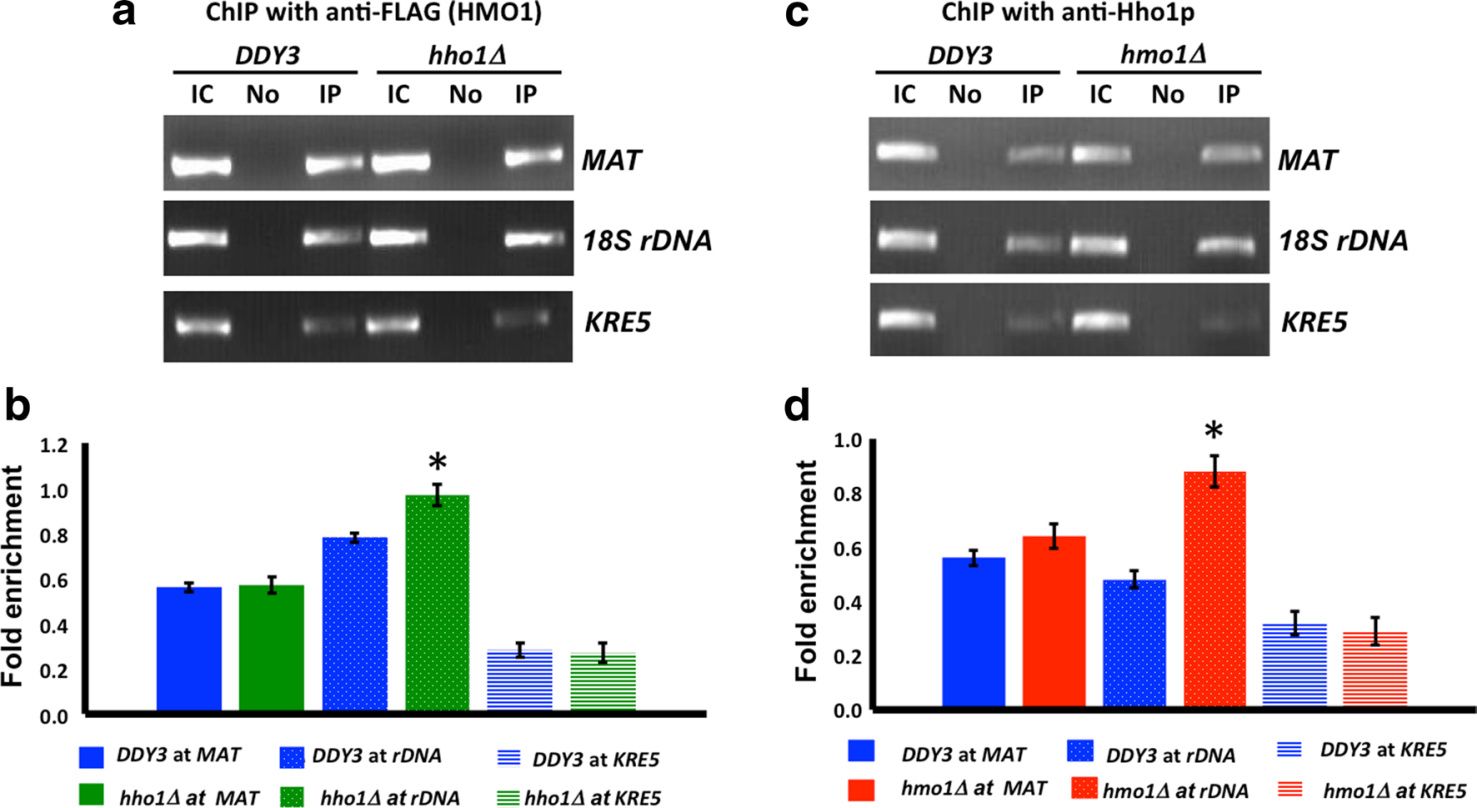
Effect of HMO1 or Hho1p on binding of the other protein. **a** Chromatin immunoprecipitation (ChIP) with *DDY3* and *hho1∆* using antibody to FLAG-tagged HMO1, monitoring binding at *MAT*, 18S rDNA, and *KRE5*. IC, input control; No, no antibody; IP, immunoprecipitation with anti-FLAG. **b** qRT-PCR analysis of ChIP data corresponding to (**a**). **c** ChIP with *DDY3* and *hmo1∆* using antibody to Hho1p, monitoring binding at *MAT*, 18S rDNA, and *KRE5*. **d** qRT-PCR analysis of ChIP data corresponding to (**c**). Data were normalized to corresponding input control at each time point. Fold enrichment = ChIP/Input DNA. Three independent experiments were performed. *Error bars* represent standard deviation. *Asterisks* represent statistical significance from *DDY3* at the same locus based on Student’s *t* test (*P* < 0.05)

To address whether the absence of either Hho1p or HMO1 influences binding of the other protein, we performed chromatin immunoprecipitation (ChIP) using antibody to FLAG-tagged HMO1 or antibody to Hho1p and monitored binding at *MAT*, 18S rDNA, and *KRE5*. As shown in Fig. 4a, b, HMO1 was enriched at *MAT* and 18S rDNA compared to *KRE5*, where only low levels were detected. Inactivation of *hho1* had no effect on HMO1 binding to *MAT* and *KRE5*, whereas modest enrichment was seen at 18S rDNA. By comparison, Hho1p was also detected both at *MAT* and 18S rDNA, with lower levels at *KRE5*; the absence of HMO1 resulted in a markedly increased association with rDNA, whereas binding to the other loci was unaffected (Fig. 4c, d). While Hho1p evidently associates with the *MAT* locus, this binding did not result in protection of linker DNA from MNase digestion in the absence of HMO1, nor was it affected by cellular levels of HMO1 (Figs. 3a, 4d); by contrast, the absence of either HMO1 or Hho1p results in a reciprocal increase in binding of the other protein at rDNA, and only elimination of both proteins renders this DNA significantly more susceptible to MNase digestion (Fig. 3d).

**Fig. 5:**
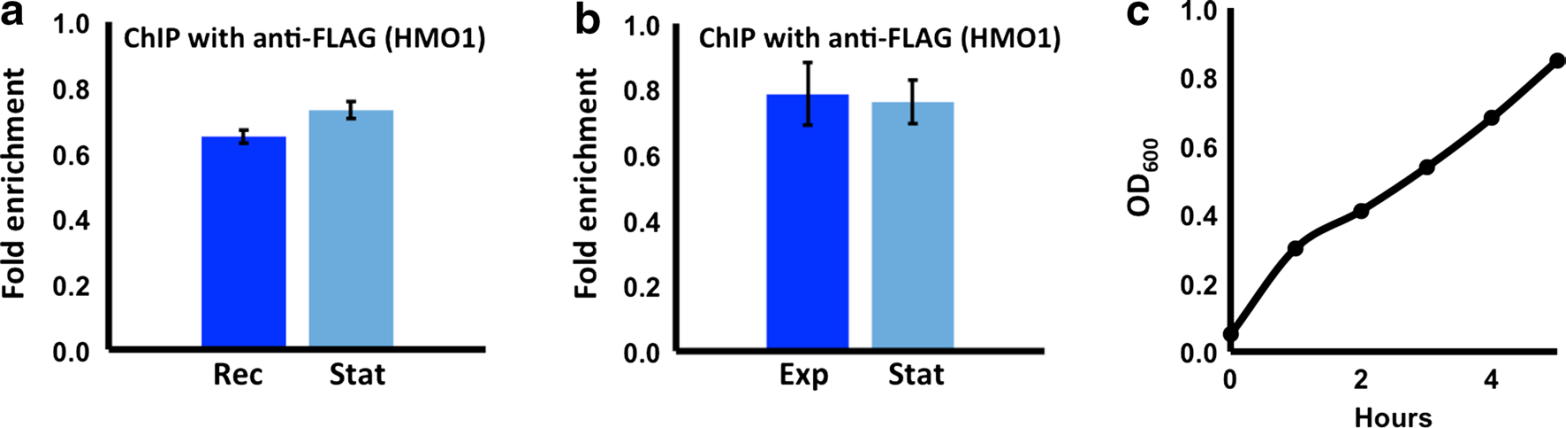
Equivalent binding of HMO1 to *MAT* in different growth phases. **a**, **b** Quantification by qRT-PCR of ChIP using antibody to FLAG-tagged HMO1 in *DDY3*, monitoring binding at the *MAT* locus. Data were normalized to corresponding input control. *Error bars* represent standard deviation of three experiments. **a** Cells recovering from quiescence (Rec) compared to stationary phase (Stat). **b** Cells in exponential phase (Exp) compared to stationary phase (Stat). Fold enrichment = ChIP/Input DNA. Three independent experiments were performed. *Error bars* represent standard deviation. **c** Growth of cells after inoculation of fresh media with stationary phase cells to OD_600_ ~0.05; cells were harvested for ChIP after 1 h (Rec) or 4 h (Exp)

In stationary phase, increased binding of Hho1p was reported to correlate with increased resistance to MNase digestion [25]. It is conceivable that such increased Hho1p binding to rDNA is responsible for residual resistance to MNase on inactivation of *hmo1*. By comparison, analysis of HMO1 binding to the *MAT* locus did not reveal markedly different levels of binding in exponential phase, stationary phase cells or cells recovering from quiescence (Fig. 5).

### Mammalian histone H1 compacts chromatin in *hmo1∆* cells

**Fig. 6:**
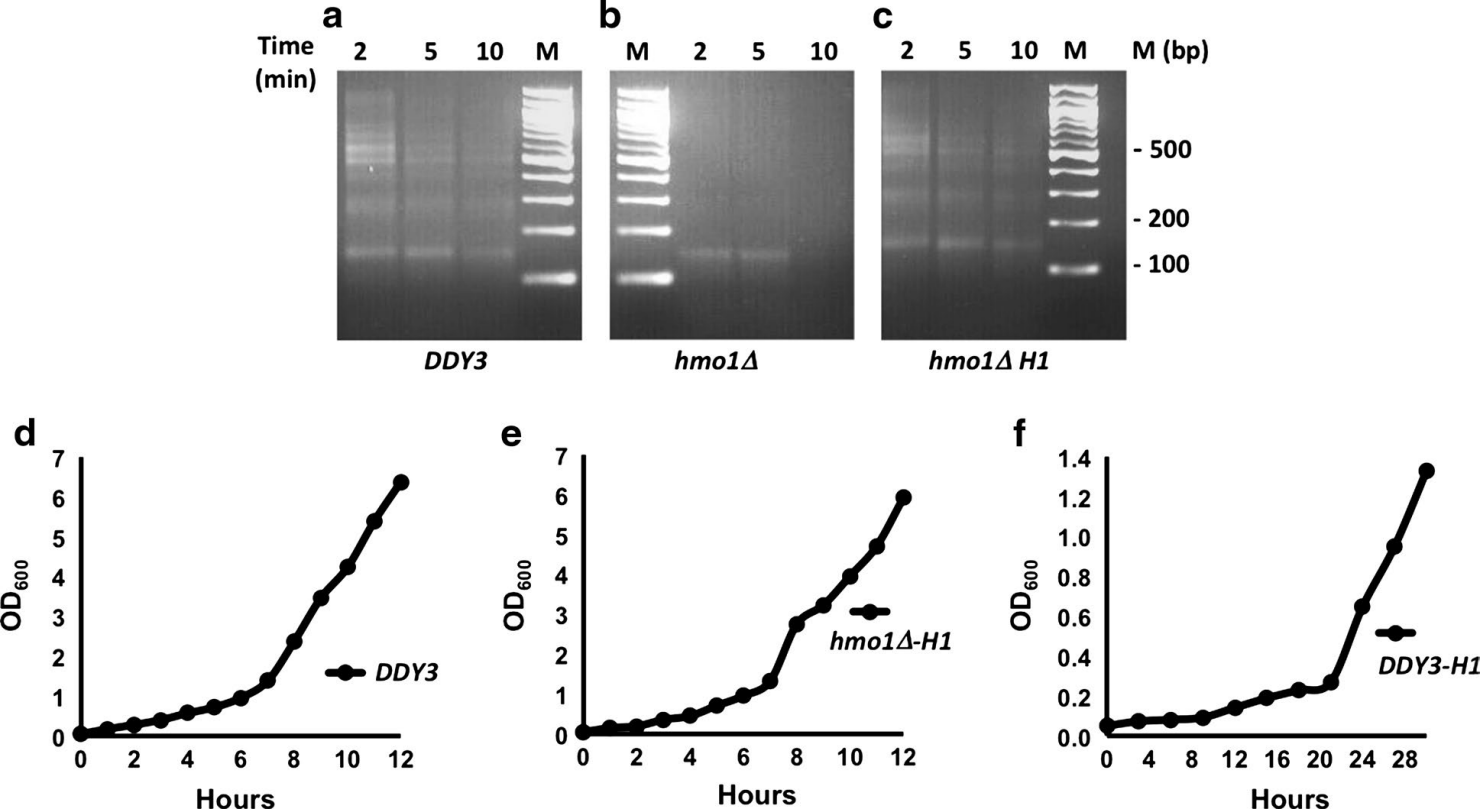
Effect of linker histone H1 on MNase sensitivity of chromatin isolated from synchronized cells and on growth rate. **a**–**c** MNase digestion of chromatin isolated from synchronized *DDY3*, *hmo1∆*, and *hmo1∆* expressing human linker histone H1.2. Cells were synchronized in G1 phase for a total of 3 h by the addition of alpha factor. Nuclei were digested with 0.25 U/µl MNase for the time indicated. Nucleosomal DNA was purified and resolved by agarose gel electrophoresis and stained with ethidium bromide. **d**–**f** Growth curve for wild-type *DDY3*, *hmo1∆* expressing H1, and *DDY3* expressing H1. Cells were grown in synthetic-defined media, and cells were collected at regular intervals to measure OD at 600 nm

Considering the conservation of core histones between species, we reasoned that the presence of heterologous histone H1 might confer resistance to MNase on yeast chromatin deleted for HMO1. A plasmid from which human histone H1.2 was constitutively expressed was transformed into wild-type, *hmo1∆* and *hmo1*-*AB* cells. Expression of histone H1.2 restored MNase resistance to chromatin isolated from *hmo1∆* cells (Fig. 1e), but not to chromatin isolated from *hmo1*-*AB* cells (Fig. 1f). This suggests that the globular domains of HMO1 are sufficient for binding to linker DNA and that HMO1-AB can compete with H1 for binding. This is similar to H1, whose globular domain binds linker DNA but does not induce compaction. The *hmo1∆* strain has a slow growth phenotype [44]. Expression of H1 in *hmo1∆* largely restores a normal growth rate, whereas *DDY3* expressing H1 grows slowly (Fig. 6d–f). We therefore performed the MNase assay on cells synchronized in G1 by the addition of alpha factor. Again, *hmo1∆* cells were more sensitive to MNase digestion, whereas *hmo1∆* cells expressing H1 exhibited a sensitivity to digestion similar to that of wild-type DDY3 (Fig. 6a).

**Fig. 7:**
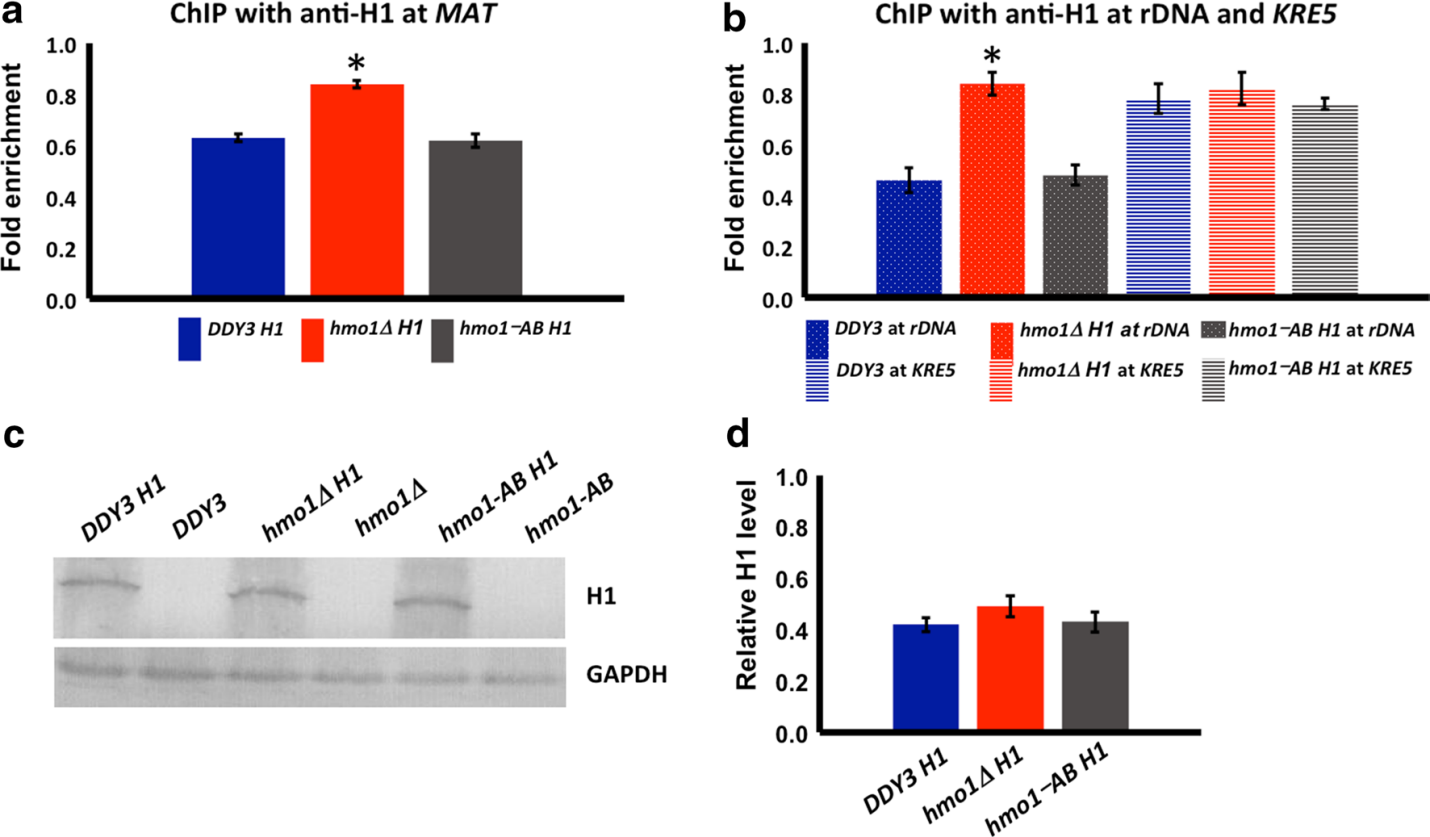
Both HMO1 and HMO1 deleted for its C-terminal tail compete with H1 for binding to chromatin. **a** Quantification by qRT-PCR of ChIP using antibody to H1 with *DDY3*, *hmo1∆*, and *hmo1*-*AB* strains, monitoring binding at the *MAT* locus. **b** qRT-PCR analysis of ChIP using antibody to H1, monitoring binding at 18S rDNA and at *KRE5*. Data were normalized to corresponding input control at each time point. Three independent experiments were performed. *Error bars* represent standard deviation. Asterisks represent statistical significance from DDY3 based on Student’s *t* test (*P* < 0.05). **c** Western blot using antibody to H1 showing equal protein level of histone H1 after transforming plasmid expressing human H1 under control of a strong, constitutive promoter in *DDY3* (*DDY3 H1*), *hmo1∆* (*hmo1∆ H1*), and *hmo1*-*AB* strain (*hmo1*-*AB H1*). Non-transformed cells *DDY3*, *hmo1∆*, and *hmo1*-*AB* were used as negative control. GAPDH expression levels were assessed in all samples as internal loading control, and the blots are representative of four independent experiments. GAPDH migrates with a Mw ~36 kDa, while H1 migrates with a Mw ~30 kDa (slower than its calculated Mw ~22 kDa). **d** Densitometric analysis of three separate blots from three independent experiments shown in (**c**). Relative H1 level = H1/GAPDH. *Error bars* represent standard deviation

ChIP using human H1.2 antibody was used to verify H1 binding to chromatin at the mating-type locus *MAT*, 18S rDNA, and *KRE5*. At the *MAT* locus, reduced H1 binding was observed in wild-type and *hmo1*-*AB* cells compared to *hmo1∆*, indicating not only direct binding of H1 to yeast chromatin, but also that both HMO1 and HMO1-AB can compete with H1 for binding (Fig. 7a). At 18S rDNA, the binding of H1 observed in *hmo1∆* was even more efficiently reduced in the presence of HMO1 and HMO1-AB (Fig. 7b). At *KRE5*, binding of H1 was equivalent in wild-type, *hmo1∆*, and *hmo1*-*AB* cells (Fig. 7b). Western blot using human H1.2 antibody verified equal cellular content of H1 in all strains (Fig. 7c, d).

### Presence of either HMO1 or histone H1 creates a chromatin environment in which repair of DNA double-strand breaks occurs with equivalent efficiency

The DNA damage response takes place within the context of chromatin. HMO1 is evicted along with core histones for repair of DNA double-strand breaks (DSBs) at the *MAT* locus, suggesting that it forms an integral part of the chromatin structure [47]. Deletion of HMO1 also appeared to generate a more accessible chromatin structure, as evidenced by faster chromatin remodeling and more efficient DNA repair in *hmo1∆* cells. Notably, *HMO1*-*AB* phenocopied the *hmo1* deletion, leading to the interpretation that the lysine-rich C-terminus is required to generate the chromatin state characteristic of wild-type cells [47].

**Fig. 8:**
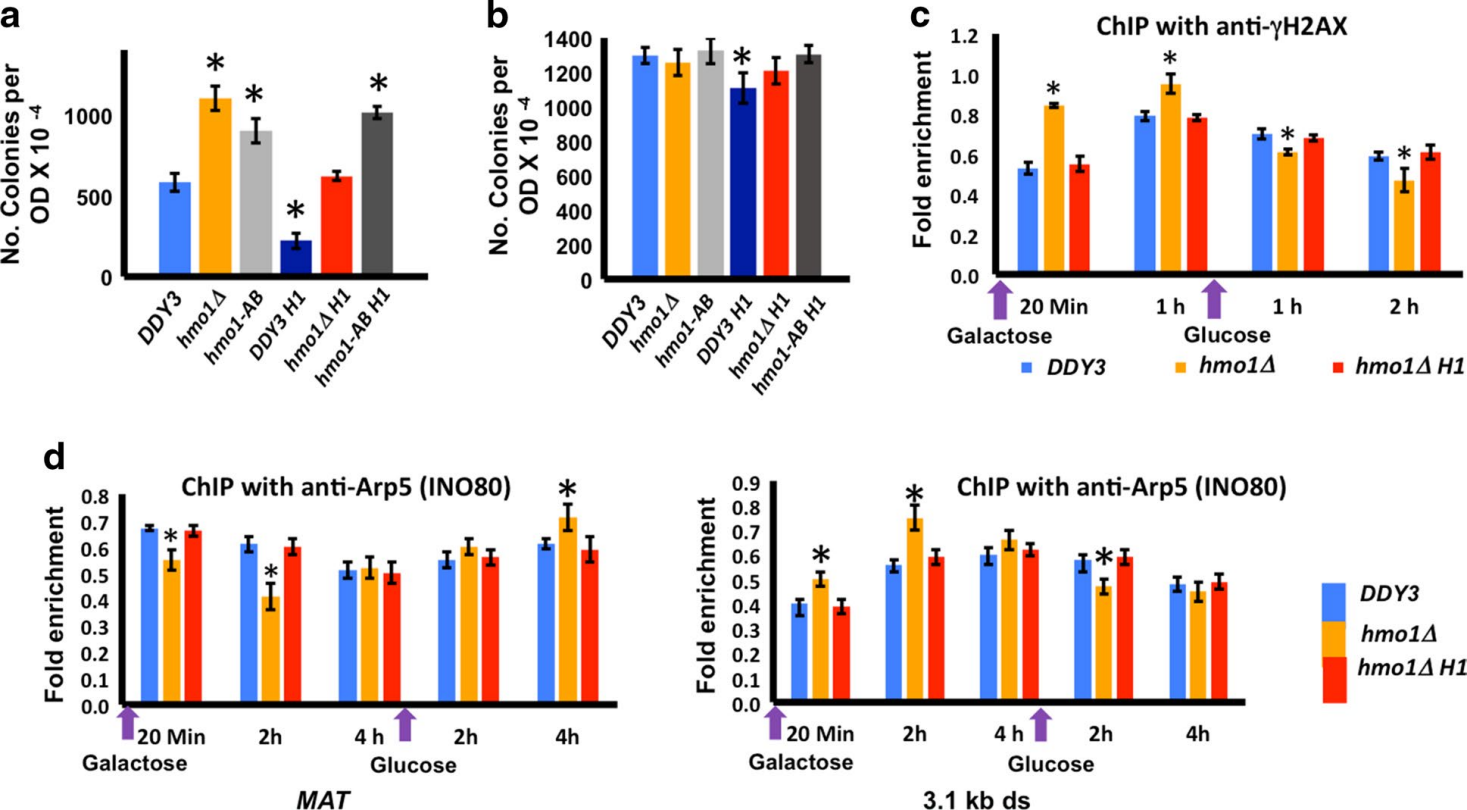
Dynamic chromatin environment in *hmo1∆* that leads to faster chromatin remodeling and DSB repair is restored to wild-type levels by expression of H1. **a** Survival of *DDY3*, *hmo1∆*, *hmo1*-*AB* and the corresponding strains expressing H1. After DSB induction, cells were plated and colonies counted. Three independent experiments were performed. *Error bars* represent standard deviation. **b** Cells not induced to express HO were plated as control. **c** qRT-PCR analysis of ChIP using antibody to phosphorylated H2A, monitoring presence of γ-H2AX at *MAT* during DNA damage (galactose) and repair (glucose). Data are normalized to corresponding input control at each time point. **d** qRT-PCR analysis of ChIP using antibody to Arp5, monitoring presence at *MAT* (*left panel*) and 3.1 kb downstream of DSB (*right panel*) during DNA damage (galactose) and repair (glucose). Data are normalized to corresponding input control at each time point. Three independent experiments were performed. *Error bars* represent standard deviation. In all panels, *asterisks* represent statistical significance from *DDY3* at the respective time points based on Student’s *t* test (*P* < 0.05)

HO endonuclease introduces a single DSB in the *MAT* locus; this DSB is repaired by a homologous recombination (HR) event that requires one of the homologous silent mating-type HM cassettes as a donor. DSBs were induced in cells harboring *HO* under control of a galactose-inducible promoter and survival assessed after plating cells with glucose to allow repair [47]. While *hmo1∆* cells more efficiently recovered from DSB induction compared to wild-type cells, this increased recovery in *hmo1∆* was reversed by expression of histone H1, as evidenced by equivalent recovery in *DDY3* cells and *hmo1∆* expressing H1 (Fig. 8a). In contrast, expression of H1 in *hmo1*-*AB* cells did not reverse the increased survival of *hmo1*-*AB* cells (Fig. 8a). The survival of wild-type cells expressing H1 was lower than wild-type cells, perhaps because of overloading cells with chromatin compacting proteins (Fig. 8a), an inference supported by the very slow growth observed for H1-expressing wild-type cells (Fig. 6f). No differences in plating efficiency were observed for cells not experiencing DSB (Fig. 8b). These observations suggest that the presence of either HMO1 or H1 creates a chromatin state in which DSB repair occurs with equivalent efficiency.

One of the earliest chromatin modification events in response to DSB is phosphorylation of histone H2A on serine 129, creating what is often referred to as γ-H2AX. This modification provides a docking site for factors such as chromatin remodeling complexes and DNA damage response proteins [48, 49]. Previously, we reported that H2A phosphorylation and dephosphorylation at the DSB site occur faster in *hmo1∆* and *hmo1*-*AB* cells compared to the isogenic wild-type parent strain, suggesting that deletion of HMO1 or its C-terminal extension results in generation of a more dynamic chromatin environment [47]. ChIP using antibody against γ-H2AX that is specific to the phosphorylated histone variant confirmed an increase in γ-H2AX in *hmo1∆* cells 20 min after DSB induction, whereas expression of histone H1 resulted in a level of H2A phosphorylation similar to that observed in wild-type cells. Following DNA repair, dephosphorylation of H2A at the damaged site was modestly faster in *hmo1∆* cells, while cells expressing either HMO1 or H1 show identical levels of H2A dephosphorylation (Fig. 8c). This suggests that the presence of H1 in *hmo1∆* reverses the more dynamic chromatin state characteristic of *hmo1∆* and *hmo1*-*AB* cells.

The chromatin remodeling complex INO80 is recruited to DSB sites in a γ-H2AX-dependent manner [50]. Association of INO80 with the *MAT* locus prior to DSB induction was also reported; this preexisting pool was suggested to be involved in *MAT* transcription, whereas γ-H2AX-dependent accumulation of INO80 downstream of *MAT* was suggested to play a role in strand invasion [51]. We monitored INO80 localization using antibody to Arp5, a conserved subunit of INO80. Upon induction of DNA damage, INO80 levels were reduced in the vicinity of the break site and instead increased 3.1 kb downstream, both events occurring faster when cells expressed neither HMO1, nor H1. This accumulation downstream of the break site was likewise reversed faster in the *hmo1∆* strain after DNA repair (Fig. 8d).

**Fig. 9:**
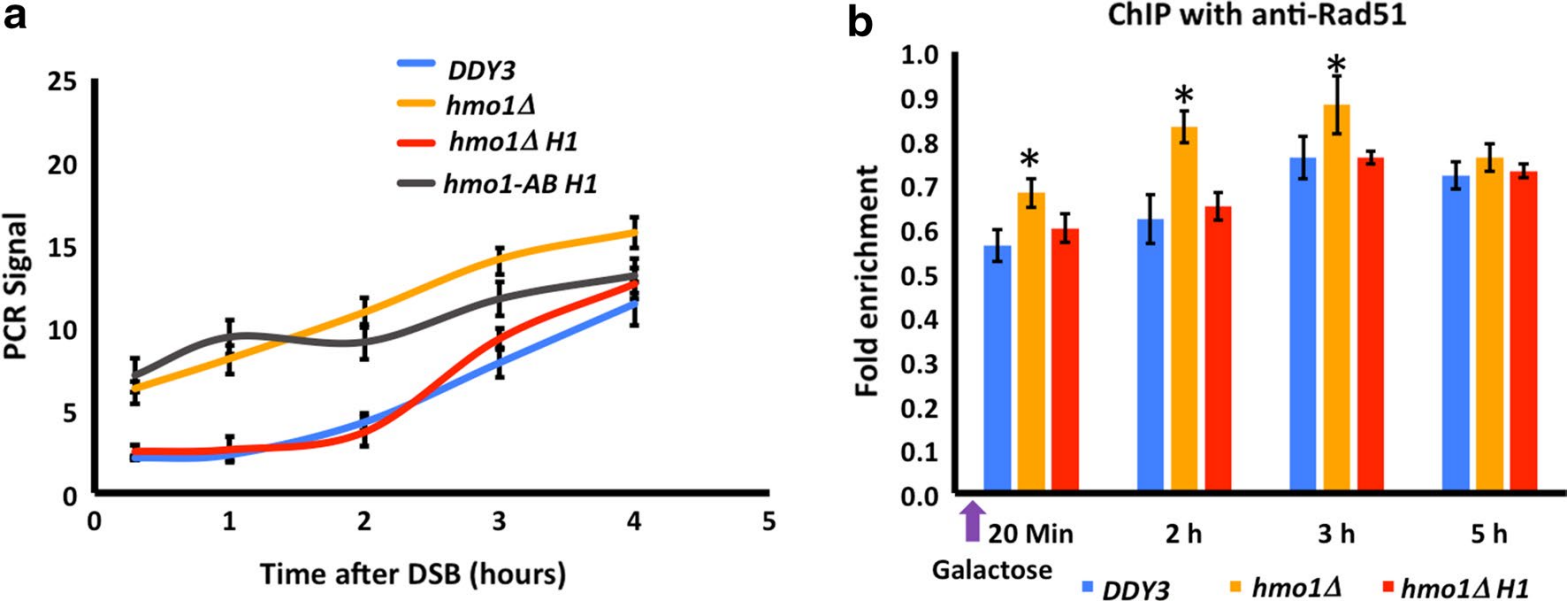
Chromatin state in *hmo1∆* that leads to faster DNA end resection and faster Rad51 recruitment after DNA double-strand break is reversed on expression of H1. **a** Quantification of DNA resection by qRT-PCR using primers that anneal 1.6 kb upstream of the DSB. PCR products were amplified after exonuclease I treatment of genomic DNA isolated at the indicated times following DSB induction. All values were normalized to that for an independent locus (*POL5*). **b** qRT-PCR analysis of ChIP using antibody to Rad51, monitoring binding at *MAT* after DSB induction (galactose). Data are normalized to corresponding input control at each time point. *Asterisks* represent statistical significance from *DDY3* at the respective time points based on Student’s *t* test (*P* < 0.05). Three independent experiments were performed

DNA end resection is required for repair of DSBs by HR, and it involves processing of the ends to yield 3′ single-stranded DNA overhangs [52]. The resected tail is the substrate for Rad51 [53]. We monitored formation of single-stranded DNA overhangs by qRT-PCR (Fig. 9a). DNA resection was faster in *hmo1∆* compared to wild-type, whereas expression of H1 in *hmo1∆* restored the slower rate of DNA end resection. *HMO1*-*AB* cells expressing H1 retained the faster rate of DNA end resection. When monitoring Rad51 recruitment to the *MAT* locus by ChIP, we observed enhanced Rad51 recruitment in the *hmo1∆* strain, whereas expression of H1 in *hmo1∆* reduced Rad51 binding to levels observed in wild type (Fig. 9b).

## Conclusion

Mammalian HMGB proteins compete with histone H1 for binding to linker DNA to create a less stable chromatin environment. Yeast HMO1 is unique among HMGB proteins in containing a lysine-rich extension, a feature also characteristic of linker histones. Our data suggest that this extension confers on HMO1 several properties of a linker histone, including resistance to MNase digestion and the generation of a chromatin environment in which events associated with DSB repair occur more slowly. This is also consistent with reported functions of other proteins containing lysine-rich repeats in condensing DNA, including the H1 proteins from protozoa that lack the globular domain as well as bacterial histone-like proteins [54, 55].

While vertebrate histone H1 has been shown to stabilize chromatin, the closest H1 homolog in *S. cerevisiae*, Hho1p, does not compact genomic DNA during vegetative growth as determined by resistance to MNase digestion (Fig. 2a; [6, 21, 22]). In stationary phase, increased binding of Hho1p correlates with increased resistance to MNase digestion, whereas analysis of HMO1 binding to the *MAT* locus did not reveal markedly different levels of binding in stationary phase cells ([47]; Fig. 5). It is conceivable that an increased level of DNA compaction and protection is necessary during stationary phase to resist environmental stress and that Hho1p contributes to this. It is also intriguing that deletion of both HMO1 and Hho1p is required for significantly increased MNase sensitivity at rDNA, while the absence of Hho1p had no effect at *MAT* and *KRE5* (Fig. 3).

We propose that HMO1 functions as a linker histone during vegetative growth, promoting a chromatin state that is also induced on expression of human H1 in *hmo1∆* cells. The equivalent phenotypes of *hmo1∆* and *HMO1*-*AB* expressing HMO1 deleted for its lysine-rich C-terminus are reversed only on expression of H1 in *hmo1∆*. This indicates that the globular domains of HMO1 compete with H1 for binding to linker DNA and that the lysine-rich extension is essential for chromatin compaction. Thus, both yeast HMO1 and H1 from higher eukaryotes possess globular domains with affinity for linker DNA connected to a lysine-rich C-terminal extension that is required for chromatin compaction.

## Methods

### Strain and plasmid construction

*DDY3* is isogenic to *W303*-*1A*. The *DDY1299* derivative of *DDY3* in which *hmo1* is deleted, strain *hmo1*-*AB*, which encodes a truncated version of HMO1 deleted for its C-terminal extension, and strain expressing HMO1-FLAG were previously described [44]. Strains *APY1* (*hho1∆*) and *APY2* (*hmo1∆hho1∆*) were constructed by transforming *DDY3*-hmo1FLAG and *DDY1299* (*hmo1∆*), respectively, with a KpnI-PmeI digest of plasmid p687 that harbors *URA3* flanked by *hho1* sequence [56]. The deletion strains were confirmed by Western blot using anti-Hho1p (ab7183; Abcam). The 2-μm plasmid pH1 containing the gene encoding human histone H1.2 under control of the strong constitutive TEF1 promoter and *LEU2* marker was synthesized by DNA2.0.

### ChIP and qRT-PCR analysis

Yeast cells were grown at 30 °C in 2 % raffinose-containing YP or in synthetic-defined (SD) dropout media to an optical density at 600 nm of 1.0. DSB was induced by the addition of galactose to a final concentration of 2 % to induce *HO*. To repress *HO* expression, 2 % glucose was added [47]. Chromatin immunoprecipitation (ChIP) was performed as described [47]. For comparison of HMO1 binding to *MAT* during exponential and stationary phase, cells were incubated in YPD medium and an aliquot (10^9^ cells) removed after 4 d (stationary phase), and the culture reinoculated into pre-warmed YPD medium and 10^9^ cells collected after 1 h (recovery from quiescence) and 4 h (exponential phase; [25]). For immunoprecipitation, the following antibodies were used: 5 µl of antibody against phosphorylated H2A (Ser129) (07-164; EMD Millipore), 2 µl of anti-Rad51 (y-180; Santa Cruz Biotechnology), 2 µl of anti-Arp5 (ab12099; Abcam), 2 µl of anti-H1.2 (ab4086; Abcam), 2 µl of anti-Hho1p (ab7183; Abcam), and 5 µl of anti-FLAG (F1804; Sigma). qRT-PCR was conducted using an ABI ViiA-7 sequence detection system and SYBR Green for detection. Data were normalized to corresponding input control at each time point. Each experiment was repeated three times, and average and standard deviations (SD) are reported. Primer sequences are available on request.

### Survival following DSB induction

A DSB was induced at the *MAT* locus by inducing expression of HO endonuclease by the addition of galactose. Survival following DSB induction was performed by plating cells on YPD or SD dropout agar media, as described [47]. Cultures to which no galactose was added were plated as a control. Each experiment was repeated three times, and data are reported as mean (±SD).

### DNA end resection

Cells were grown at 30 °C to an OD_600_ of 1.0, and DSBs were induced by the addition of 2 % galactose. Cells were harvested after various induction times, and genomic DNA was extracted by vortexing cells with glass beads and phenol. Twenty microliters of genomic DNA (60 ng in 1X Exonuclease I buffer (New England Biolabs)) was digested with 20 units of *E. coli* exonuclease I at 37 °C overnight. The level of DNA resection adjacent to the specific DSB was measured by qPCR using primers annealing 1.6 kb upstream of the DSB. All values were normalized to values for an independent locus on chromosome 5 (*POL5*). The assay was repeated three times and reported as mean (±SD) [47].

### Micrococcal nuclease (MNase) assay

Yeast cells were grown at 30 °C in 2 % glucose-containing YP or in synthetic-defined (SD) dropout media to an optical density at 600 nm of 0.8. To synchronize yeast cells in G1 phase for a total of 3 h, α-factor (10 µg ml^−1^; Zymo Research) was added as described [57]. One ml culture was removed to prepare spheroplasts, followed by nuclei isolation by using EZ Nucleosomal DNA Prep Kit (Zymo Research). Nuclei were treated with 0.25 U/µl of micrococcal nuclease. Reactions were stopped after 2, 5, or 10 min, and pure nucleosome DNA was isolated and subsequently resolved in 2 % agarose gels. Nucleosomal DNA was also probed by PCR at loci enriched for HMO1 (18S rDNA, *MAT*, 0.2 kb upstream of *MAT*) and a locus at which HMO1 was not detected (*KRE5*). Primer sequences were previously reported [47] or are available on request.

### Western blot

Cells were grown at 30 °C to an OD_600_ of 0.8. Fifty microliters of culture was removed to extract protein. Cells were lysed by vortexing with glass beads using lysis buffer (100 mM Tris–HCl, 300 mM NaCl, 2 mM EDTA, 10 % glycerol, 5 % Triton X-100) containing 100 mM β-mercaptoethanol, 0.2 mM phenylmethylsulfonyl fluoride (PMSF), and protease inhibitor cocktail tablet (Roche). Protein concentration was measured using BCA Protein Assay Kit (Pierce). Fifteen micrograms of protein was resolved on 12 % SDS-PAGE, and the resolved proteins were transferred to polyvinylidene fluoride membrane. Anti-histone H1.2 (ab4086; Abcam), anti-Hho1p (ab7183; Abcam), and anti-FLAG (F1804; Sigma) were added at a 1:1000 dilution, whereas secondary antibody was added at a dilution of 1:5000. As internal loading control, anti-GAPDH (ab9485; Abcam) was added at a 1:5000 dilution. The blots were developed by using CN/DAB substrate kit (ThermoFisher). The intensity of immunoreactive bands was determined using image J software for densitometric analysis.

### Growth curve

A single colony was inoculated into 6 ml YP or synthetic-defined media containing 2 % glucose and cultured overnight at 30 °C. After overnight incubation or when cell reached log phase, cells were diluted to an OD_600_ of 0.05 in 25 ml culture volume. OD_600_ was recorded at regular intervals.

## Abbreviations

ChIP: chromatin immunoprecipitation
MNase: micrococcal nuclease
